# Informed self-assessment versus preceptor evaluation: a comparative study of pediatric procedural skills acquisition of fifth year medical students

**DOI:** 10.1186/s12909-020-02221-2

**Published:** 2020-09-21

**Authors:** Muhammed Elhadi, Hazem Ahmed, Ala Khaled, Wejdan K. Almahmoudi, Samah S. Atllah, Ahmed Elhadi, Hamida Esahli

**Affiliations:** 1 Faculty of Medicine, University of Tripoli, Tripoli, 13275 Libya; 2 Department of Pediatrics, Faculty of Medicine, University of Tripoli, Tripoli, Libya

**Keywords:** Education, Medical student education, Self-assessment, Simulation, Pediatrics

## Abstract

**Background:**

Simulation training is widely used in medical education as students rarely perform clinical procedures, and confidence can influence practitioners’ ability to perform procedures. Thus, this study assessed students’ perceptions and experiences of a pediatric skills program and compared their informed self-assessment with their preceptor-evaluated performance competency for several pediatric clinical procedures.

**Methods:**

A total of 65 final-year medical students attended a weeklong pediatric skills training course by the University of Tripoli that used a manikin and various clinical scenarios to simulate real-life cases. Participants completed questionnaires self-assessing their performance skills, while examiners evaluated each students’ competency on five procedural skills (lumbar puncture, nasogastric tube insertion, umbilical vein catheterization, intraosseous access, and suprapubic aspiration) using an objective structured clinical examination (OSCE) model. Differences between agreement levels in question responses were evaluated through a nonparametric chi-square test for a goodness of test fit, and the relationship between confidence levels and the OSCE scores for each procedure was assessed using Spearman’s rank-order correlation.

**Results:**

All participants completed the informed self-assessment questionnaire and OSCE stations. The frequency differences in agreement levels in students’ questionnaire responses were statistically significant. No significant differences were found between students’ self-assessment and preceptors’ evaluation scores. For each procedure’s passing score rate, umbilical vein catheterization had the highest passing rate (78.5%) and nasogastric tube placement the lowest (56.9%). The mean performance scores were above passing for all procedures. The Wilcoxon signed-rank test revealed no significant differences between participants’ self-assessment and their preceptor-evaluated competency; students correctly perceived and assessed their ability to perform each procedure.

**Conclusions:**

High competence in several life-saving procedures was demonstrated among final-year medical students. The need for consistent and timely feedback, methods to increase medical students’ confidence, and further development and improvement of competency-based assessments are also highlighted.

## Background

Clinical rotations are designed to provide medical students with the necessary knowledge, practical experience, and skills before commencing their medical practice. The final year of medical school is a particularly critical time for students to acquire clinical and practical skills, both procedural and cognitive, to ensure greater competency [1, 2]. The University of Tripoli has introduced a new skill lab in pediatric clerkship in 2017 for final-year medical students, based on simulation training and manikins. This university offers Bachelor of Medicine and Bachelor of Surgery (MBBCh) programs, equivalent to the medical doctor degree (MD), which comprise a pre-clinical year, five clinical years, and a clinical training internship year. The university’s skill lab was designed to provide students with the advanced skills and knowledge necessary for their internships after completing medical school, as part of a new educational curriculum introduced recently to meet international standards and ensure high-quality care and patient safety in Libya.

Simulation training is widely used in medical education because it allows programs to teach clinical skills in a safe learning environment, which is essential in pediatrics training, as students rarely get the chance to perform clinical procedures. Simulation training can also improve safety and reduce costs and harm to patients. Students and early-career doctors can effectively develop professional health skills, knowledge, and attitudes through this method [3–6].

Assessment of the learning and education process is essential to ensure students are adequately trained to perform procedures in a hospital. Additionally, the relationship between students’ practicing confidence (based on informed self-assessment) and their performance competency (based on preceptor evaluation) is critical [7–9]. Confidence can influence practitioners’ ability to perform a procedure, and patients may be at greater risk of adverse events if a practitioner is incompetent or performs inaccurately [10]. In 1999, Kruger and Dunning demonstrated that people with incompetent skills may overestimate their ability to perform certain tasks and fail to recognize their own misperception [11]. The only way to ensure physicians acquire the skills necessary for their work is to improve their ability to accurately appraise their own performance and measure their abilities, thereby ensuring their optimal and competent treatment and management of patients [7, 12, 13].

Informed self-assessment is a process that includes learners in assessing whether or not learner-identified expectations have been achieved [14]. In education, self-assessment allows learners to recognize their strengths and weaknesses both during and after the learning process [15]. In healthcare practice, self-assessment allows practitioners to identify their strengths and weaknesses and react accordingly to improve the services provided. For example, an important aspect of medical practice is avoiding potential risks by recognizing the need for help or support, or declining to perform certain actions due to awareness of inadequate abilities. This awareness allows medical practitioners to act appropriately, without hesitation or negligence.

Previous studies have addressed the relationship between informed self-assessment and preceptor evaluation in different clinical skills. Some have suggested that self-evaluation tools based on specific assessments can more accurately determine an individual’s competency [16–18]. Several have noted that self-assessment is an inaccurate measurement of competence [19, 20]. However, few studies have systematically examined the relationship between student self-assessments and preceptor evaluations. No guidance based on national or international consensus exists on methods of assessing medical students’ skills as part of the learning outcomes using their informed self-assessment as a predictor of preceptor-evaluated competency.

Therefore, this study evaluated the relationship between students’ informed self-assessments (i.e., their confidence in their learning outcomes) and preceptors’ competency evaluations (i.e., students’ ability to accurately perform the trained procedures) following completion of a pediatric skills training course. The fundamental principle of accurate self-assessment, as reflected by students’ self-assessment of their learning outcomes in the pediatric skills training course, should be applicable to other clinical skills.

### Aim

To assess students’ perceptions and experiences of a pediatric clinical skills program and to compare their informed self-assessment with their preceptor-evaluated performance competency for several pediatric clinical procedures, including lumbar puncture, intraosseous infusion, nasogastric tube placement, umbilical vein catheterization, and suprapubic aspiration.

## Methods

The study sample, enrolled in August 2017, comprised 65 final-year medical students who attended a special pediatric skills training course. Students who undertook the course were informed about each part of the course and were provided with an introductory summary lecture about the course structure and learning outcomes expected by the course’s end. The weeklong, comprehensive course, introduced by the University of Tripoli, used a manikin and various clinical scenarios to simulate real-life cases. Each clinical scenario was first devised by a professor with two instructors; then, the students were allowed to voluntarily perform a certain skill under the supervision of the instructors, who provided feedback about any mistakes or misunderstandings. The goal was to help students acquire crucial pediatric life support skills that would be necessary during clinical rotations in their foundation (internship) year.

**Fig. 1:**
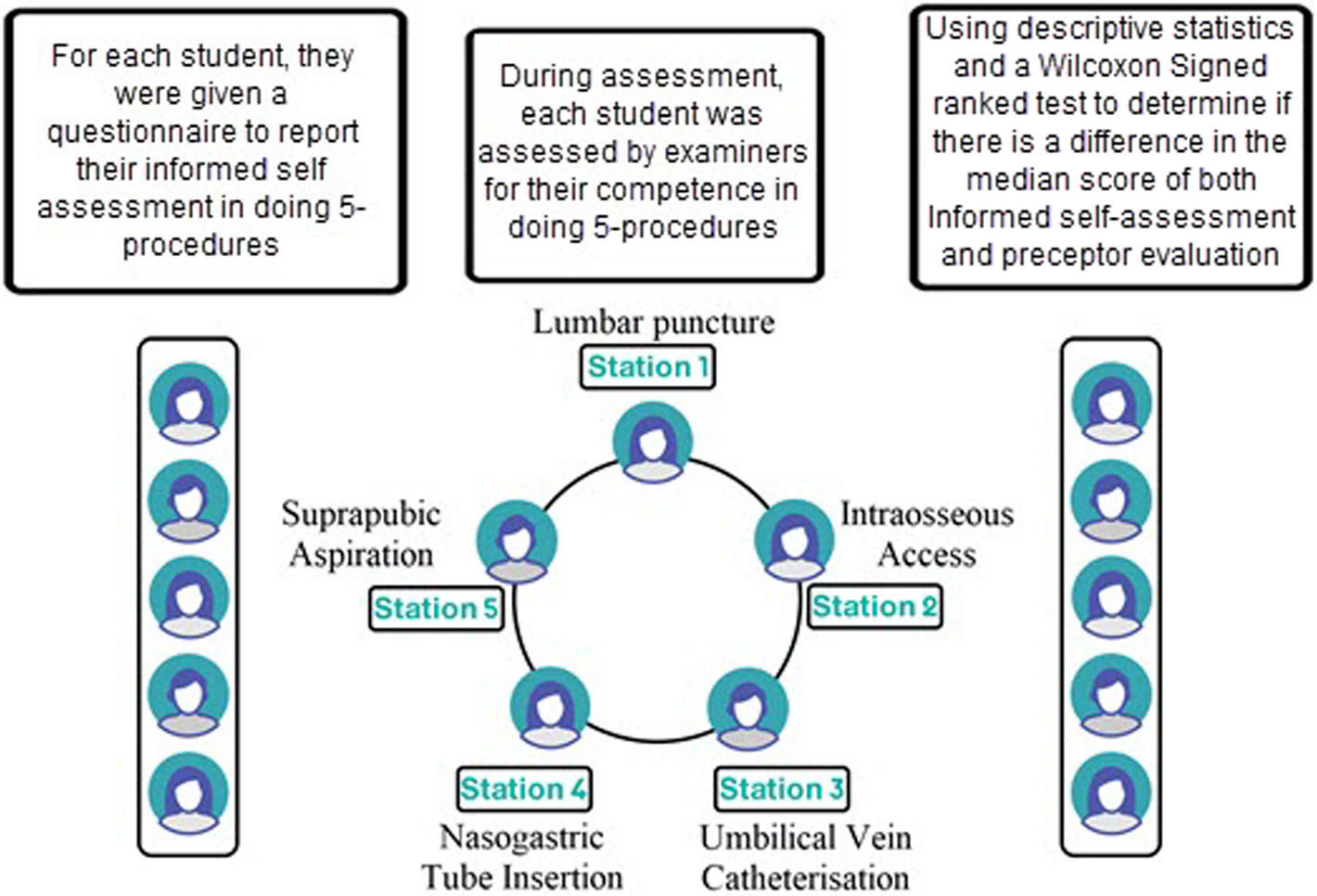
Methodological steps of the study

Participants completed informed self-assessment of their skills using a questionnaire. Self-assessment was defined as the involvement of participants in judging whether or not learning expectations were met or whether their ability to perform procedures were of an acceptable standard [14]. For each of the five procedures, the participants rated their confidence in their abilities. Subsequently, their actual competency in each procedure was assessed based on preceptor evaluations using an objective structured clinical examination (OSCE) model. Figure 1 illustrates the two main steps of this study.

### Participant questionnaires and self-assessment

Participants were first provided with a validated questionnaire to collect feedback on the course. This questionnaire included seven items assessed on a 4-point Likert scale (1 = *strongly disagree*, 2 = *disagree*, 3 = *agree*, 4 = *strongly agree*), and its internal consistency and reliability were assessed using Cronbach’s alpha [21].

The researchers then explained the informed self-assessment and evaluation procedure to the participants and provided them with another questionnaire to assess their confidence in performing five procedures (i.e., lumbar puncture, nasogastric tube placement, umbilical vein catheterization, intraosseous infusion, and suprapubic aspiration). Each procedure was covered during the weeklong pediatric skills course, and participants were able to practice in low- and medium-fidelity simulations. All items were rated on a 4-point Likert scale (1 = *not confident enough to perform even under supervision*, 2 = *not confident enough to perform without supervision*, 3 = *confident enough to perform without supervision*, 4 = *confident enough to teach the procedure*).

### Preceptor competency evaluation

Following participants’ self-assessment, their competency in each of the five procedures were assessed by examiners through an OSCE. For each procedure, a checklist was created that contained discrete steps derived from the existing university curriculum. These checklists were pre-validated using a pilot sample of students, and to ensure reliability, these underwent several revisions by the clinical department, and finally by the head of the medical education department. The pilot assessment was performed by medical students and pediatric demonstrators unaware of the present study design or research question. To evaluate inter-rater reliability of the assessments completed during the main study, two examiners were present at each station during the test.

In the OSCE, a 10-min time limit and specific schedule was set for each task. At each station, participants were presented with a standardized scenario requiring the use of each particular procedure, and had to then perform the procedure using manikins and prosthesis parts. Two examiners evaluated each participant and rated their performance on a 4-point Likert scale (4 = *excellent*, 3 = *good*, 2 = *pass*, 1 = *fail*). Overall scores were also calculated for each participant at each station based on the accumulated total of correct OSCE checklist steps (see the supplementary files for the content of each procedure’s checklist).

All participants provided informed written consent and were blinded to the study design and objectives. The OSCE checklists and self-assessment questionnaires were not made available to participants before the start of the study. For the OSCE evaluations, two sets of the five stations were set up, allowing 10 participants to complete the test at the same time. To prevent measurement bias, participants who had completed the assessment were not allowed contact with those still waiting to participate. All assessments were conducted by pediatric skill demonstrators and pediatricians who volunteered to take part in the project and were blinded to the research questions, informed self-assessment results, and study outcomes. The examiners were not involved in the analysis or interpretation of the data following the evaluations. All were trained and had previous experience conducting pediatric OSCE exams or serving as demonstrators in clinical skills education. All evaluations were completed on a single day.

After the OSCE evaluations were completed, participants’ competency levels were compared with their self-assessed confidence. To define their competency level, each participant received a raw score based on the OSCE checklist. Each skill had a specific OSCE score; possible scores for each were: lumbar puncture 0–27, intraosseous infusion 0–32, nasogastric tube 0–25, umbilical venous catheterization 0–26, and suprapubic aspiration 0–24. This score was matched against a score from 1 to 4 provided by both examiners at each station to determine the overall performance (excellent, good, average, poor). A score of at least 60% was defined as passing for each OSCE station.

### Statistical analysis

First, participants’ feedback on the pediatric skills course was tabulated against the questionnaire response rate. Descriptive statistics were used to calculate the mean and standard deviation for continuous variables and frequency and percentage for categorical variables. Responses of *strongly disagree*, *disagree*, *agree*, and *strongly agree* were coded as 1, 2, 3, and 4, respectively. Differences between agreement with each item were calculated using a nonparametric chi-square test for goodness of test fit for a single sample. The response rate was calculated as a percentage. The internal consistency of the instrument was determined using Cronbach’s alpha [21].

A Wilcoxon matched-pairs signed-rank test was used to test the hypothesis that the distribution of difference scores between informed-self assessment and preceptor evaluation score was symmetric about zero. Spearman’s rank-order correlation was conducted to assess the relationship between participants’ informed self-assessment level and OSCE score for each procedure. *P*-values less than 0.05 were considered statistically significant. All analyses were conducted using IBM SPSS version 25.

## Results

All 65 participants completed both the informed self-assessment questionnaire and all OSCE stations. The Cronbach’s alpha was 0.71 for the self-assessment questionnaire and 0.827 for the OSCE checklists, indicating high reliability and good internal consistency.

Table 1 illustrates students’ responses concerning the pediatrics course evaluation. Statistically significant differences in the frequency of participants who indicated various levels of agreement were observed (*p* < .001).

**Table 1:** Frequency of participants’ response to different questions concerning the pediatric course evaluation

Item	Level of agreement				Mean	Standard division	*p*-value
	Strongly disagree	Disagree	Agree	Strongly agree			
The instructors were enthusiastic.	0 (0%)	6 (9.2%)	43 (66.2%)	14 (21.5%)	4.00	0.79	< 0.001
The instructors were well prepared.	2 (3.1)	2 (3.1%)	37 (56.9%)	24 (36.9%)	4.28	0.67	< 0.001
The skills/procedures taught were appropriate for fifth-year medical students.	4 (6.2%)	8 (12.3%)	36 (55.4%)	17 (26.2%)	3.95	0.90	< 0.001
Assigned readings/videos/materials helped prepare me to perform the procedure/skills.	6 (9.2%)	1 (1.5%)	22 (33.8%)	36 (55.4%)	4.26	1.16	< 0.001
Feedback on my performance during the sessions was helpful.	11 (16.9%)	14 (21.5%)	23 (35.4%)	17 (26.2%)	3.71	1.04	0.183
I think this course will benefit me in my intern (Imtiaz) year.	8 (12.3%)	3 (4.6%)	30 (46.2%)	24 (36.9%)	3.95	1.24	< 0.001
Overall, the course was educationally worthwhile.	1 (1.5%)	11 (16.9%)	35 (53.8%)	18 (27.7%)	4.08	0.71	< 0.001

**Fig. 2:**
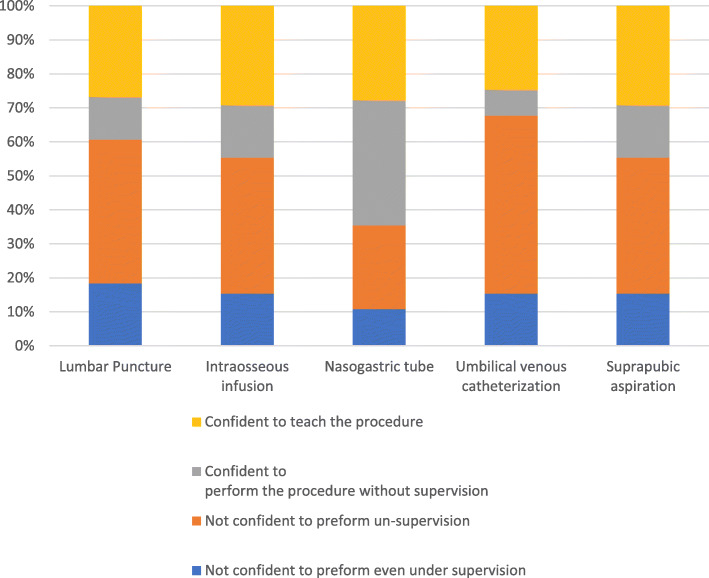
Percentage of student pre-test informed self-assessment

**Fig. 3:**
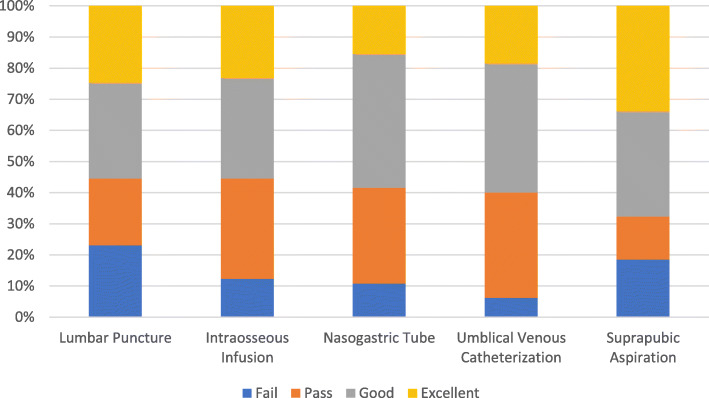
Percentage of students’ skills scores according to preceptors’ evaluation

Preceptor evaluations revealed varying levels of competency among the participants. Figure 2 and Fig. 3 illustrate the results of their informed self-assessment and preceptor evaluation scores for each clinical procedure. No significant differences were found between self-assessment and preceptor evaluation scores; the latter scores were thus very similar to students’ self-rated assessments. The passing score rates for each procedure were higher than expected, with umbilical vein catheterization having the highest passing rate (78.5%) and nasogastric tube placement having the lowest (56.9%). Meanwhile, the mean performance scores were above passing for all procedures. Table 2 presents the passing scores and passing rates for each procedure.

**Table 2:** Skill type, total scores, and passing scores

	Lumbar puncture	Intraosseous infusion	Nasogastric tube	Umbilical venous catheterization	Suprapubic aspiration
Total score	27	32	25	26	24
Passing score	16.2	19.2	15	15.6	14.4
Mean score (SD)	18.29 (5.30)	21.17 (4.99)	16.17 (2.93)	17.94 (3.31)	15.88 (4.04)
Passing rate n (%)	39 (60%)	44 (67.7%)	37 (56.9%)	51 (78.5%)	42 (64.6%)
Minimum score	9	9	8	9	4
Maximum score	27	30	22	25	21

The Wilcoxon signed-rank test revealed no significant differences between the participants’ self-assessment and their preceptor-evaluated competency (Table 3). The null hypothesis that the distribution of difference between informed self-assessments and preceptor evaluation scores would be symmetrical about zero was accepted—the students’ confidence in their skills matched their preceptor-evaluated competence. Thus, the participants were able to correctly perceive and assess their ability to perform each procedure. However, the results also showed that the participants with lower confidence and self-assessed abilities demonstrated higher competence and received higher preceptor evaluations, despite their negative perceptions.

**Table 3:** Differences between medical students’ self-assessment with preceptors’ evaluation of competency assessed by OSCE

	Medical student self-assessment	Preceptor evaluation		
Procedural skill	Mean (standard deviation)	Mean (standard deviation)	Wilcoxon’s Z	*p*-value
Lumbar puncture	2.62 (1.03)	2.57 (1.10)	−0.28	0.978
Nasogastric tube	2.82 (0.97)	2.63 (0.87)	−1.161	0.246
Umbilical vein catheterization	2.42 (1.03)	2.72 (0.84)	−1.853	0.64
Intraosseous infusion	2.62 (1.03)	2.66 (0.97)	−0.192	0.848
Suprapubic aspiration	2.58 (1.07)	2.80 (1.16)	−1.032	0.302

Spearman’s rank-order correlation was used to assess the relationship between perceived self-assessment levels and OSCE scores for each procedure. A preliminary analysis showed the relationship to be monotonic, as assessed by visual inspection of a scatterplot. However, there was no statistically significant correlation between the confidence level and OSCE performance score recorded for the students as follows: lumbar puncture *rs*(63) = − 0.193, *p* = 0.123, nasogastric tube insertion *rs* (63) = 0.009, *p* = 0.944, umbilical vein catheterization *rs* (63) = − 0.006, *p* = 0.959, suprapubic bladder aspiration *rs* (63) = − 0.155, *p* = 0.219, and intraosseous access *rs* (63) = 0.007, *p* = 0.954).

## Discussion

This study illustrates the use of comparing informed self-assessment with faculty evaluation of students as an outcome measure for pediatric procedural skill acquisition. Despite the difficulty and complexity of the procedures assessed in this study, passing rates were high, reflecting that most participants had received instruction and constructive feedback, and hence, gained practical experience. Statistically, participants’ informed self-assessment scores were largely similar to their preceptor evaluation scores, with no significant difference between the results of self-assessment and those of faculty assessment for a given competence.

This study’s findings contradict several previous studies that found no agreement between confidence and competence, specifically those of Donoghue et al. on pediatric OSCE skills [22] and Barnsley et al. on junior doctors in their first internship year [23]. A similar study of house officers in the United Kingdom found no significant correlation between performance and self-assessment [24]. In a study of third-year medical students in the United States, in which students’ self-assessments of 10 skills were compared to evaluation scores given by trained simulated patients, Isenberg et al. found significant differences between the students’ and patients’ scores for clinical skills, but not procedural skills [25]. The students were less confident in their procedural competencies. Finally, a study of podiatric medical students found no statistically significant correlation between academic performance and clinical self-assessment, either at the start of students’ third year or after completion of their study [26]. In other words, students’ self-assessment of their clinical performance was not associated with their academic performance. We expected that students would not perform exactly in line with their confidence level, as there was some concern about their ability to perform the procedures correctly and well. We consider that the students who did not achieve a passing score or demonstrated lower competence were those who did not actually perform the procedures on the manikin during the training course, depending instead on learning by watching or doing only discrete steps. It is crucial to fully understand these results and implement this knowledge in future educational and training programs for medical students.

Overall, the ability to objectively evaluate one’s performance and skills allows healthcare workers to achieve a successful balance between engaging in everyday medical practice and ensuring patient safety. Medical students’ perceptions must be considered, as social and personal factors continuously affect learners’ use of both structured and informal learning and assessment practices by the learners, which could help inform their self-assessment of their performance [27]. This highlights the need to develop faculty programs to raise awareness of the value of self-assessment.

There is no gold standard for the assessment of medical students’ confidence and competency levels. Therefore, the validity of these assessment tools in their current form was determined through consultation with the pediatric educational department, several discussions and revisions, and pilot testing.

Some of the medical students who participated in this study were unfamiliar with all the steps of certain procedures, such as lumbar punctures and suprapubic aspiration, and omission of vital steps lead to failure in these students’ evaluations. However, higher success was observed in procedures such as umbilical vein catheterization and nasogastric tube placement, perhaps due to students’ previous experience performing these procedures during the training course. The variation observed in students’ performance might reflect their experiences or engagement during the training course, as some students performed procedures multiple times, whereas others performed them only once or not at all, instead, only observing. Insufficient training or lack of opportunity to practice the procedure prior to the evaluation could explain the variations in this study’s results.

A possible source of bias in this study’s results could be the examiners. However, all examiners were blinded to the study design and objectives, and were not aware that the test was for research purposes. All were either instructors with previous experience in conducting pediatric OSCE exams or pediatricians. To prevent subjective or personal bias, two examiners were assigned to each station, both of whom independently assessed and scored the participants’ performance. The medical students in this study were evaluated in a simulation-based environment using manikins. Although they had gained some experience performing advanced procedures usually conducted by senior pediatricians, they had little to no real experience managing clinical situations in pediatric intensive care units, either alone or under supervision by junior pediatricians. Thus, the results may overestimate students’ abilities, which were evaluated based on training with a manikin rather than human beings. Regardless, this study provides evidence for faculty and future classes supporting the benefits of the pediatric skills training course. Additionally, the results demonstrate alignment between students’ competence and their own confidence in their skills, unlike many previous studies. This could indicate that students correctly assessed their competence in these procedures, or that the examiners overestimated their performance.

Reliable and standardized testing methods are necessary to assess the performance and competency of medical students and junior doctors. To some extent, OSCEs and simulation-based educational courses can meet this demand. However, it is also essential to develop an educational approach that allows students to accurately assess their competence and abilities.

No significant relationship between preceptor evaluations and informed self-assessment was found among this study’s sample of final-year medical students after completing the pediatric intensive care skills course. Continued training and improved self-assessment will thus be vital to properly ensure their ability to perform these procedures. This may imply preparing a lifelong educational curriculum designed to enhance students’ and practitioners’ pediatric intensive care skills.

Before accepting the results of this study, it is critical to consider any potential bias or error that may have affected the outcomes. However, the findings indicate that medical students can perform complex procedures and demonstrate a high standard of care with relatively high confidence in their skills.

## Conclusions

This study demonstrates the comparability of student self-assessment results with those of faculty assessments for several pediatric clinical procedural skills among final-year medical students and supports the implementation of the University of Tripoli’s pediatric skills training course as a standard curriculum. The need for constructive feedback, methods to increase medical students’ confidence, and further development and improvement of competency-based assessments are also highlighted.

## Data Availability

The datasets used in this study are available from the corresponding author on reasonable request.

## Abbreviation

OSCE: objective structured clinical examination
